# Differential variation patterns between hubs and bottlenecks in human protein-protein interaction networks

**DOI:** 10.1186/s12862-016-0840-8

**Published:** 2016-12-01

**Authors:** Erli Pang, Yu Hao, Ying Sun, Kui Lin

**Affiliations:** 1 MOE Key Laboratory for Biodiversity Science and Ecological Engineering, College of Life Sciences, Beijing Normal University, No 19 Xinjiekouwai Street, Beijing, 100875 China; 2 Beijing Key Laboratory of Gene Resource and Molecular Development, College of Life Sciences, Beijing Normal University, Beijing, 100875 China

**Keywords:** Protein interaction network, Hubs, Bottlenecks, Variation rate

## Abstract

**Background:**

The identification, description and understanding of protein-protein networks are important in cell biology and medicine, especially for the study of system biology where the focus concerns the interaction of biomolecules. Hubs and bottlenecks refer to the important proteins of a protein interaction network. Until now, very little attention has been paid to differentiate these two protein groups.

**Results:**

By integrating human protein-protein interaction networks and human genome-wide variations across populations, we described the differences between hubs and bottlenecks in this study. Our findings showed that similar to interspecies, hubs and bottlenecks changed significantly more slowly than non-hubs and non-bottlenecks. To distinguish hubs from bottlenecks, we extracted their special members: hub-non-bottlenecks and non-hub-bottlenecks. The differences between these two groups represent what is between hubs and bottlenecks. We found that the variation rate of hubs was significantly lower than that of bottlenecks. In addition, we verified that stronger constraint is exerted on hubs than on bottlenecks. We further observed fewer non-synonymous sites on the domains of hubs than on those of bottlenecks and different molecular functions between them.

**Conclusions:**

Based on these results, we conclude that in recent human history, different variation patterns exist in hubs and bottlenecks in protein interaction networks. By revealing the difference between hubs and bottlenecks, our results might provide further insights in the relationship between evolution and biological structure.

**Electronic supplementary material:**

The online version of this article (doi:10.1186/s12862-016-0840-8) contains supplementary material, which is available to authorized users.

## Background

Proteins rarely function in isolation but rather interact with other genes or proteins to form a complex network to carry out certain biological functions. Protein-protein interaction (PPI) networks are thus crucial for the understanding of protein functions [1], protein evolution [2] and related diseases [3]. In the past decade, researchers have identified many PPI networks across different species [4–8]. These networks were reported to be scale-free [9–11], i.e., the degree of a node follows the power law distribution. The majority of nodes links to only few other nodes; very few nodes link to a large number of other nodes.

There are many measures to describe the topology of a network. The degree of a node, namely, the number of nodes to which the node connects, is a basic local index. In a network, nodes of high degree are called hubs, on which several studies have been conducted. Earlier studies mainly focused on the correlation between node degree and functional importance because hubs are hypothesized to be more biologically important. They found that hubs are encoded by essential genes [12, 13]. The relationship between evolutionary conversion and node degree was then investigated. The negative correlation between evolutionary rate and node degree has been reported [14–20].

Another most elementary global network topology measure is betweenness, the frequency with which a node lies on the shortest path between other nodes [21]. Proteins with a high level of betweenness are called bottlenecks. Several studies have been conducted on bottlenecks. For example, it was found that bottlenecks tend to be encoded by essential genes [13, 22] and that a negative correlation exists between evolutionary rate and betweenness [18, 19]. In addition, proteins of high betweenness are more likely to be of older evolutionary age than those of low betweenness [23].

The relationship between drug target and degree, as well as the relationship between drug target and betweenness was investigated. It was found that proteins known to be a drug target have higher degree and/or betweenness values than an average protein [24]. When comparing hubs with bottlenecks at the level of protein evolutionary age in a yeast PPI network, hubs were found to depend more on the protein evolutionary age than bottlenecks do [25].

The relationship between degree and betweenness was discussed. Previous research studies found a positive relation between the degree of a protein and its betweenness [22, 26]. This is to say that overlapping proteins may exist between hubs and bottlenecks. Once protein redundancy is accepted, then several questions can be addressed. For instance, how many overlapping proteins are there, and do differences exist between hubs and bottlenecks? Additionally, what are the variation patterns of hubs and bottlenecks, and are there any differences between them? With the development of sequencing technology, more human genome sequences are available that provide us with the opportunity to analyse them. To address these questions, we compared hubs and bottlenecks using genome-wide variation amongst human populations and four protein-protein interaction datasets. Because certain proteins overlap between them, we think that only the difference between their own special proteins can represent their differences. Therefore, we retained hub-non-bottleneck nodes and non-hub-bottleneck nodes and compared them. In addition, we removed the influence of incomplete network and the cut-off of node degrees/betweenness. Furthermore, we attempted to determine whether other factors affect our results. Taken together, we conclude that different variation patterns exist between hubs and bottlenecks in human protein interaction network. Our result highlights the relationship between hubs and bottlenecks in protein-protein networks and help in understanding the evolution of proteins.

## Results

### Combined protein-protein interactions

To analyse a large number of PPIs, we first integrated the existing PPIs. We extracted the physical PPIs from BioGRID [27], DIP [28], HPRD [29] and IntAct [30]. Protein IDs represented in each database were mapped to Ensembl transcript IDs [31] using the common idmapping files. There were 151,810 interactions among 14,617 proteins in BioGRID, 1595 interactions among 1514 proteins in DIP, 35,305 interactions among 9069 proteins in HPRD, and 3060 interactions among 2313 proteins in IntAct, respectively. After removing duplicate interactions, we merged these four data sets. A total of 167,795 different physical interactions among 15,714 proteins were retained.

In this network, we examined the degree distribution, p(k), which is the frequency of the proteins interacting with k other proteins, and found that the distribution of degree followed the power law distribution (*R* ^2^ = 0.84) (Additional file 1: Figure S1). This result indicated that the network was a scale-free network, i.e., few proteins interacting with many other proteins and many proteins interacting with few other proteins, which was consistent with previous studies [10, 11].

### The negative correlation between the variation rate of a protein and its degree or betweenness

Previous research has shown a negative correlation between the degree of a protein and its rate of variation [14–20]. To determine whether such a correlation exists in recent human history, we calculated the degree of each protein using the R package and obtained the variation rate of proteins using PAML [32]. There was a weak negative association between the variation rate and degree of protein (Spearman’s ρ = −0.20, *P* < 2.2 × 10^−16^). There are also reports of negative correlation between the betweenness of a protein and its rate of variation [18, 19]. To examine this relationship, the betweenness of each protein was obtained using the R package. We also found a weak negative correlation between the variation rate and betweenness (Spearman’s ρ = −0.14, *P* < 2.2 × 10^−16)^. This finding indicates that proteins with a high degree or betweenness tend to change slowly, which is in agreement with the previously mentioned interspecies studies.

**Fig. 1:**
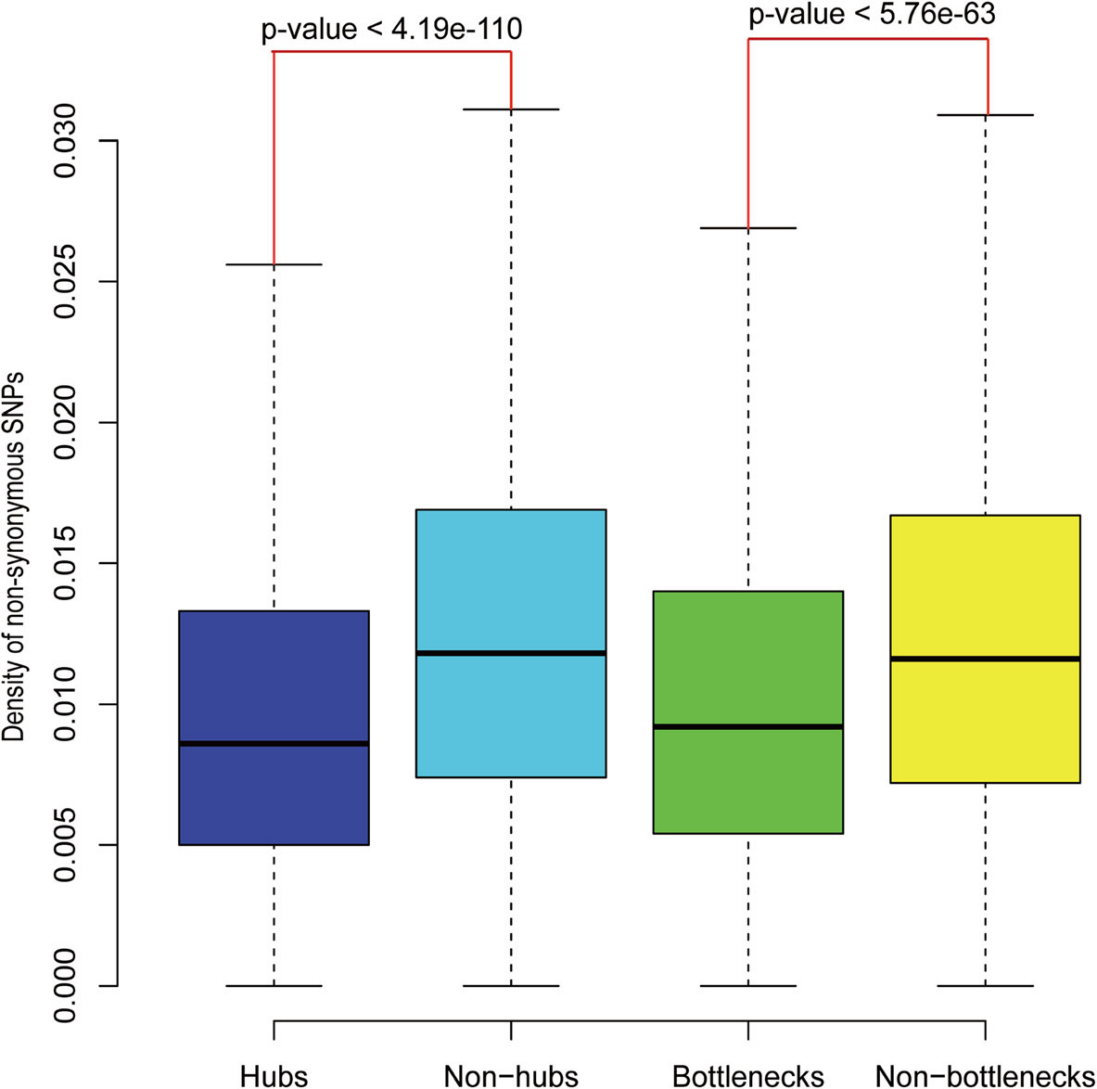
Distribution of the variation rate of proteins in human protein-protein networks

We further compared the variation rates of hubs with those of non-hubs. As illustrated in Fig. 1, the hubs changed significantly more slowly than non-hubs (0.00860 vs 0.01180, Mann-Whitney U test: *p* < 4.19 × 10^−110^). For the bottlenecks and non-bottlenecks, the pattern was similar (Fig. 1, 0.0092 vs 0.0116, Mann-Whitney U test: *p* < 5.76 × 10^−63^).

### Hubs tending to be bottlenecks

**Fig. 2:**
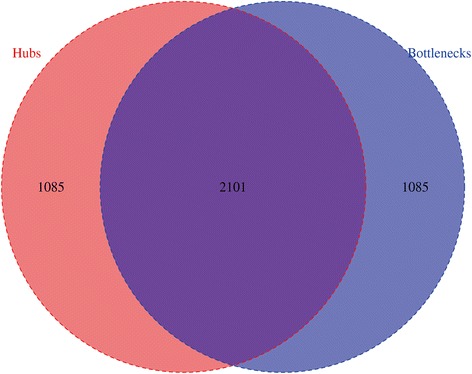
Venn diagram of the number of hubs and bottlenecks

Previous studies have identified a positive correlation between the degree of a protein and its betweenness [22, 26]. In this network, we also found such a significant positive correlation (Spearman’s *ρ* = 0.76, *p* < 2.2 × 10^−16^). However, it was unclear how many overlapping nodes there were between hubs and bottlenecks. To address this, we identified hubs and bottlenecks using the top 20% of the corresponding distribution. We found that hubs tend to be bottlenecks (Fig. 2).

### Slower variation of hubs than that of bottlenecks

**Fig. 3:**
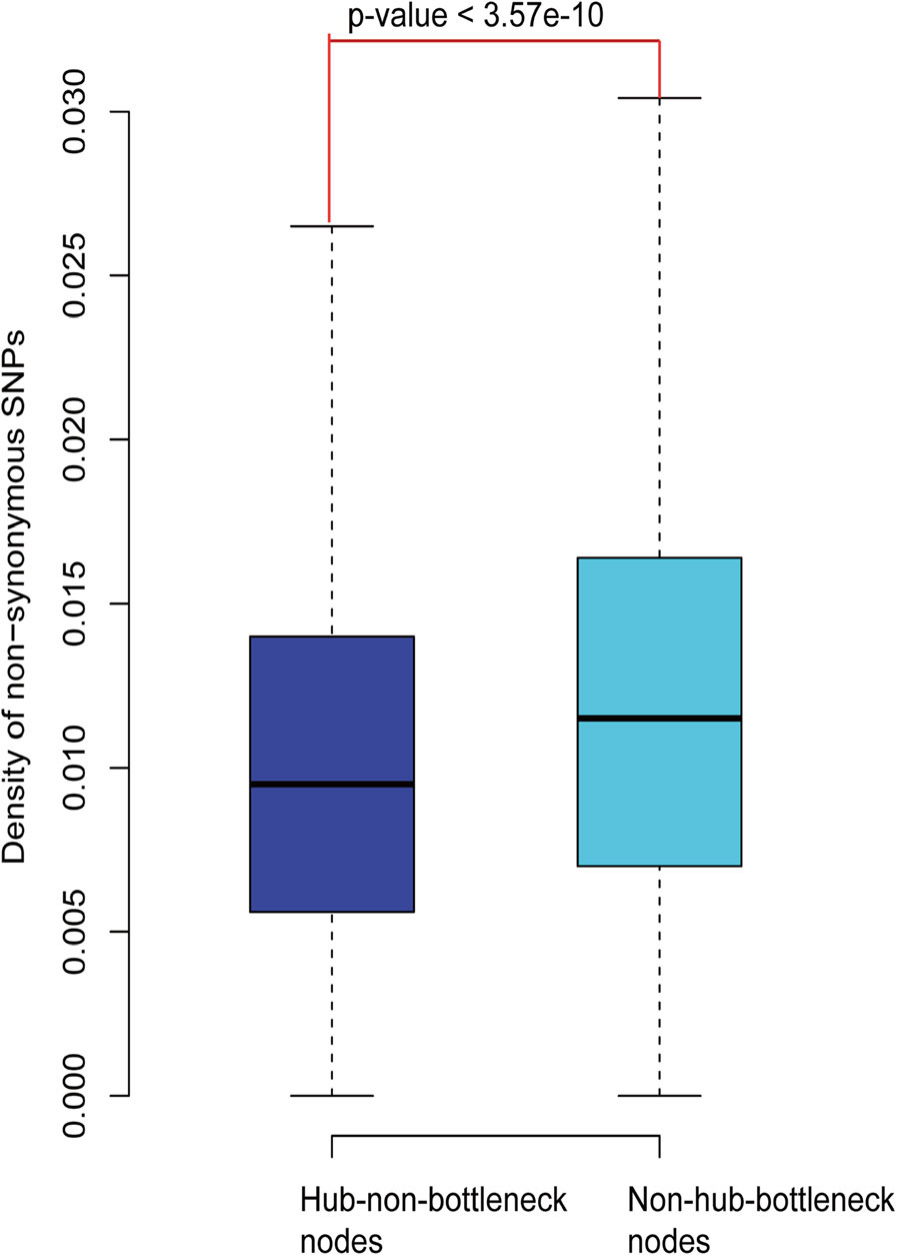
Distribution of the variation rate of hub-non-bottlenecks and non-hub-bottlenecks

There are very large overlaps of proteins between hubs and bottlenecks. Therefore, it is necessary to distinguish between the hubs and bottlenecks, otherwise we would not know whether proteins are hubs or bottlenecks when the proteins are important. We further portioned them into three categories as shown in a previous study [22]: hub-bottlenecks, hub-non-bottlenecks, and non-hub-bottlenecks. To distinguish between hubs and bottlenecks, we removed the overlapping nodes that are hub-bottlenecks and retained their own special nodes: hub-non-bottlenecks and non-hub-bottlenecks. These two sets of proteins would help us better identify the differences between the hubs and bottlenecks. We compared the variation rate between them. As described in Fig. 3, the variation rate of hub-non-bottleneck nodes is significantly lower than that of non-hub-bottleneck nodes (0.00950 vs 0.01150, Mann-Whitney U test: *p* < 3.57 × 10^−10^). Additionally, we used the protein-coding genetic variations in 60,706 humans [33] to redo our analysis, and the result was similar (0.1398 vs 0.1511, Mann-Whitney U test: *p* < 6.45 × 10^−9^; Additional file 2: Figure S2).

Although we integrated human physical protein-protein interactions from four public databases, the number of interactions is likely to be much larger than the current data suggest [24]. To remove the potential influence of an incomplete network, we first randomly selected 80% of the nodes from the human PPIs and reconstructed the PPI network. This randomization process was carried out 1000 times, and the analysis repeated for 1000 randomized networks. The different variation patterns between hubs and bottlenecks were all observed. We then randomly selected 80% of the edges from the human PPIs and repeated the randomization process as the randomized nodes and the different variation patterns between hubs and bottlenecks were still observed. This process suggested that the incompleteness of the PPI network might not have influenced our results.

Protein evolution is a complex process. In this process, many factors might affect the variation rate of proteins, such as the essentiality, expression breadth, expression levels and topologies of the network [34, 35]. We found different variation rates between hubs and bottlenecks. We then addressed whether differences exist between hub-non-bottlenecks and non-hub-bottlenecks when the above factors are taken into account. First, we estimated the difference for the enrichment of essential genes and found that there was no significant difference in the enrichment of essential genes between hub-non-bottlenecks and non-hub-bottlenecks (Fisher’s exact test: *ρ* = 0.92, *p* = 0.45). We then analysed the expression breadth and the expression levels of these genes. For the expression width, both of their medians were 15. For the expression levels, there were a significant difference between hubs and bottlenecks (4.15 vs 1.94, Mann-Whitney U test: *p* < 2.66 × 10^−13^). Using K-means, we clustered the hubs and bottlenecks by expression levels and focused on one subgroup where there were 846 hub-non-bottlenecks and 1038 non-hub-bottlenecks. No significant difference was observed between the expression level of hubs and that of bottlenecks (2.61 vs 1.94, Mann-Whitney U test: *p* = 0.27). Furthermore, we found that the variation rate between them differed (0.009800 vs 0.01160, Mann-Whitney U test: *p* < 6.22 × 10^−8^, Additional file 3: Figure S3). These analyses implied that the difference between hubs and bottlenecks remained when excluding the effects of essentiality, expression breadth and expression levels. These results demonstrated that hub-non-bottlenecks change significantly more slowly than no-hub-bottlenecks.

### Stronger constraint on the hubs than on the bottlenecks

**Fig. 4:**
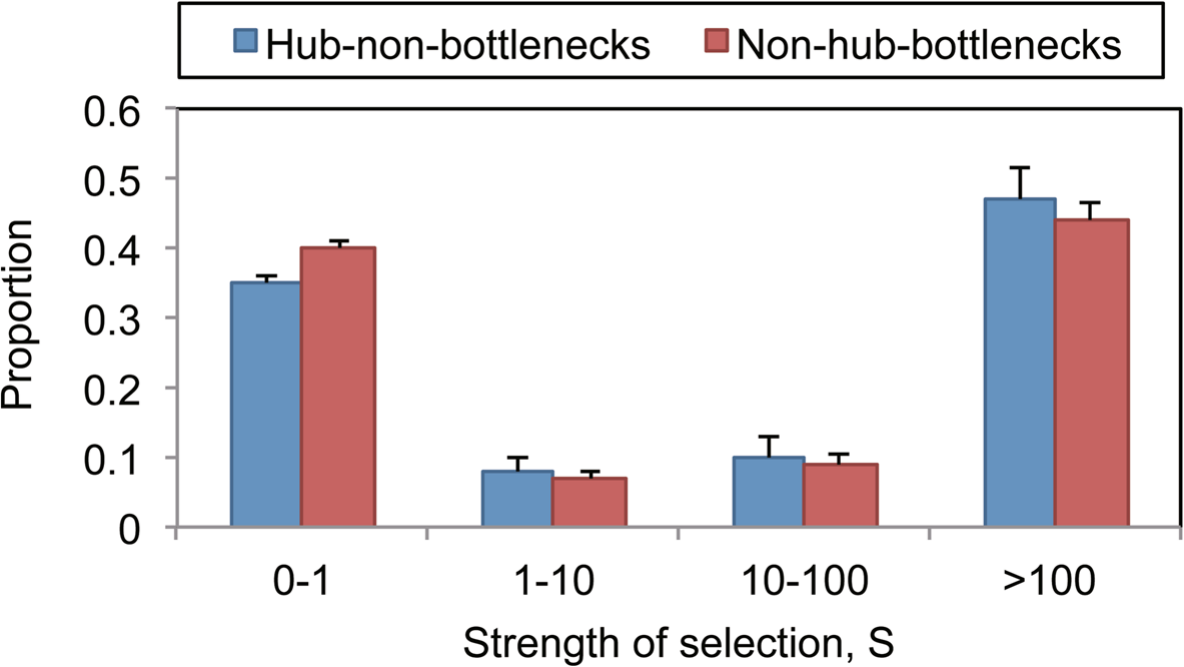
Distribution of the strength of selection on non-synonymous SNPs in hub-non-bottlenecks and non-hub-bottlenecks. Error bars denote SE around the estimated proportions

Based upon the above analysis, we found only the different variation rate between hubs and bottlenecks. However, we do not know whether there is different constraint on them. To answer this question, we used the likelihood-based method [36] to infer the gamma distribution of fitness effects of hub-non-bottles and non-hub-bottlenecks. The advantage of this method is that it can control for demographic effects. Because some mutations at CpG sites are much more frequent than at other sites, we excluded the CpG-related SNPs to control the effect of these SNPs. The sharp parameters of hub-non-bottlenecks and non-hub-bottlenecks were 8.87e-2 (6.87e-2, 1.47e-1) and 7.57e-2 (6.25e-2, 1.06e-1), respectively. The mean strength of selection acting on hub-non-bottlenecks and non-hub-bottlenecks were 1.62e+5 (8.18e+2, 7.63e+5) and 1.23e+5 (3.02e +3, 4.72e+5), respectively. The proportion of mutations falling within these four categories of S values reflects different strengths of selection on both hub-non-bottlenecks and non-hub-bottlenecks (Fig. 4). We found that hub-non-bottlenecks exhibited a lower fraction of mutations with |S| < 1 (34.9%) than that of non-hub-bottlenecks (39.5%). This result indicated that the strength of selection on the hub-non-bottlenecks was stronger than that on the non-hub-bottlenecks.

### Fewer non-synonymous sites on domains of hubs than on those of bottlenecks

We next estimated the non-synonymous sites on domains of hubs. We found that there were significantly fewer non-synonymous SNPs in the domains of hub-non-bottlenecks than in random proteins (*p*-value = 0.02, see Methods). However, for the non-hub-bottlenecks, we did not observe a similar pattern. We further compared the non-synonymous SNPs in domains of hub-non-bottlenecks with those of non-hub-bottlenecks. There were significantly fewer non-synonymous SNPs in the domains of hub-non-bottlenecks than those of non-hub-bottlenecks (Fisher’s exact test: ρ = 0.83, *p*-value < 2.2 × 10^−16^). We next analysed the functions of hub-non-bottlenecks and non-hub-bottlenecks. PANTHER tool (version 10.0) on the web server (http://geneontology.org) was used to analyse GO molecular function [37]. The top 5 significantly overrepresented GO terms, which must be located in more than 3 layers in the structure of GO, are listed in Tables 1 and 2 for hub-non-bottlenecks and non-hub-bottlenecks, respectively. As shown in these tables, hub-non-bottlenecks may participate in nucleic acid binding, whereas non-hub-bottlenecks may be involved in signal transduction.

**Table 1:** Molecular function enrichment for hub-non-bottlenecks

GO term	P-value
Nucleic acid binding	4.78e-79
Organic cyclic compound binding	5.62e-74
RNA binding	3.03e-68
Poly(A) RNA binding	3.00e-64
DNA binding	1.89e-11

**Table 2:** Molecular function enrichment for non-hub-bottlenecks

GO term	P-value
Receptor binding	7.78e-15
Signalling receptor activity	1.03e-14
Transmembrane signalling receptor activity	1.87e-13
G-protein coupled receptor activity	2.38e-07
Cell adhesion molecule binding	5.15e-06

## Discussion

In this study, we identified different variation patterns between hubs and bottlenecks based on four human protein-protein interaction databases and the genetic variation of 1092 human genomes. Based on the hypothesis that a protein will change more slowly over time if it is an important protein, we can infer from our analysis that hubs may be more important than bottlenecks in human protein-protein interaction network. It is anticipated that our results will help to further understand protein evolution and highlight the relationship between hubs and bottlenecks.

Our results were based on protein-protein interaction networks and genome-scale variation, such that the analysis would be affected by both their quality and coverage. To construct a comprehensive protein-protein interaction network, we used four databases: BioGRID, DIP, HPRD, and IntAct. However, BioGRID was heavily over-represented in the final dataset. This may bias the results by any latent factor associated with BioGRID. We repeated the calculation on the HPRD dataset. It exhibited similar results (0.00900 vs 0.01020, Mann-Whitney U test: *p* < 7.31 × 10^−4^; Additional file 4: Figure S4).

In this study, both the hubs and bottlenecks are in the top 20% of the corresponding distribution. To remove the influence of the cut-off of hubs and bottlenecks, we varied the cut-off from 5 to 40% as previously reported [22] and found that the cut-off almost had no significant impact on our results (Additional file 5: Figure S5).

We found that hub-non-bottlenecks may participate in nucleic acid binding. One example is Sin3A-associated protein, 18 kDa (SAP18, ENST00000382533), which plays a key role in the regulation of eukaryotic gene expression. SAP18 is a binding partner of mammalian tribbles homologue 1 (a human locus, TRIB1), which has been shown to significantly impact plasma lipid. The knockdown of the Sap18 in mouse liver decreased plasma lipid levels [38]. This example showed that hub-non-bottlenecks play an important function through nucleic binding.

## Conclusions

We used an integrated protein-protein network to evaluate the difference between hubs and bottlenecks at the level of mutation in recent human history. We found different evolution patterns between hubs and bottlenecks, which showed no differences in the enrichment of essential genes, expression breadth, and expression levels between hubs and bottlenecks. Although we cannot conclude that the topology of the network is the key factor in the difference between hubs and bottlenecks, we did demonstrate the relationship between the variation rate and topology of the human protein-protein network. Based on our results, we believe that the bottlenecks are not as important for a general understanding and may be mainly data driven in the protein-protein network as previously argued by Barabasi [39].

## Methods

### Data sources

In this study, we mainly used six types of data: genome sequence, genome annotation, genome-wide variation from human populations, protein-protein interactions, essential genes, and RNA-Seq reads.

The human genome sequence was based on the February 2009 *Homo sapiens* assembly, GRCh37, downloaded from Ensembl [31] (http://asia.ensembl.org/index.html). The models of the protein-coding genes were retrieved from version 16 (April, 2013) of the GENCODE project [40], whose aim was to annotate all evidence-based gene features in the human genome. Genome-wide variations were from two datasets: the genome-wide set of genetic variations among 1092 human genomes [41] downloaded from the 1000 Genomes Project (http://www.internationalgenome.org), and the protein-coding genetic variation in 60,706 humans [33] downloaded from Exome Aggregation Consortium (ftp://ftp.broadinstitute.org/pub/ExAC_release/current/, release 0.3.1).

The protein-protein interactions were from four databases: BioGRID (BIOGRID-ORGANISM-Homo_sapiens-3.3.122.tab2.txt) [27], DIP (Hsapi20150101.txt) [28], HPRD (BINARY_PROTEIN_PROTEIN_INTERACTIONS.txt) [29], and IntAct (intact.txt) [30] (Table 3). For BioGRID, we extracted only the interactions that met three conditions: (1) “Organism Interactor A” is “9606”, (2) “Organism Interactor B” is “9606”, and (3) “Experimental System Type” is “physical”. For DIP, we collected the following interactions: (1) “Taxid interactor A” is “taxid:9606(Homo sapiens)”, (2) “Taxid interactor B’ is “taxid:9606(Homo sapiens)” and (3) “Interaction type” is “physical interaction” or “direct interaction”. For HPRD, we filtered the self-interactions. For IntAct, we retrained the following interactions: “Type(s) interactor A/B” is “protein” and “Interaction type” is “direct interaction”. We then compiled the non-redundant human protein-protein interactions as the union interactions. While identifying non-redundant interactions, we converted the protein IDs from each database to Ensembl_TRS [31] using the idmapping file available at ftp://ftp.uniprot.org/pub/databases/uniprot/current_release/knowledgebase/idmapping/idmapping.dat.gz.

**Table 3:** List of protein-protein interaction databases used in this study

Database name	Number of interactors	Number of interactions	Release date/version	Uniform resource locator
BioGrid	19,128	269,778	March, 2015, Release 3.3.122	http://thebiogrid.org
DIP	4298	6464	January 1, 2015	http://dip.doe-mbi.ucla.edu/dip/Main.cgi
HPRD	9617	39,240	April 13, 2010, Release 9	http://www.hprd.org
IntAct	83,772	523,070	February 23, 2015	http://www.ebi.ac.uk/intact/

A list of essential genes was obtained from DEG 11.0 [42] (http://www.essentialgene.org), which collects essential genes from the literature [43].

### Definition of hubs and bottlenecks

We defined hubs as the proteins that are in the top 20% of the degree distribution. We also defined bottlenecks as the proteins that are in the top 20% of the betweenness distribution. The degree and betweenness of a protein in human protein-protein interactions were calculated using the R package igraph [44]. We used the command “degree(graph)” for degree, and “betweenness(graph,directed = FALSE)” for betweenness.

### Variation rate of proteins

We used the density of non-synonymous SNPs (dN), which is the number of non-synonymous substitution per non-synonymous site, to measure the variation rate of a protein. For human genes, sequences that change over time can be identified according to the number of SNPs present. The dN values of human genes were then estimated by applying PAML (version 4.8) based likelihood method [32] on the two sequences.

### Inference of the strength of selection acting on hub-non-bottlenecks and non-hub-bottlenecks

The program DoFE v3.0 proposed by Eyre-Walker et al. [36] was implemented to infer the strength of selection. The software can be downloaded from http://www.lifesci.sussex.ac.uk/home/Adam_Eyre-Walker/Website/Software.html. Some mutations at CpG sites occur much more frequently than at other sites. To control for the effect of CpG-related SNPs, we excluded all CpG-related SNPs as previously described [45].

### Randomization process

To test the significance of the percentage of non-synonymous SNPs on the domains of hubs and bottlenecks (called observed percentage), we followed a randomization method. We produced 10,000 randomized datasets each containing K proteins, where K is the number of hub-non-bottlenecks or non-hub-bottlenecks. For each randomized dataset, we calculated the percentage of non-synonymous SNPs on the domains of K proteins. The proportion of the randomized datasets with a lower percentage of non-synonymous SNPs on the domains of K proteins compared to the observed percentage in the 10,000 randomized datasets is a direct estimation of the p-value that can be attached to the hypothesis that non-synonymous SNPs on domains have a similar percentage.

### Protein domain identification

We downloaded pfam_scan.pl from Pfam (http://pfam.xfam.org) [46]. The Pfam database is a large collection of protein families, each represented by multiple sequence alignments and hidden Markov models [46]. In Pfam28.0, there are 16,230 protein families. We executed the program with default parameters.

### Processing RNA-Seq reads

To estimate the expression level and width of genes as defined below, we downloaded paired-end RNA-Seq reads from 16 different tissues. These data are available at ftp.sra.ebi.ac.uk/vol1/fastq/ERR030/ (ERR030872~ERR0887). Reads were assessed for quality and trimmed using Trimmomatic version 0.35 [47]. Reads smaller than 25 bp were excluded. We excluded reads from brain tissue because less than 50% of reads were retained. Therefore, we analysed only 15 tissues. Trimmed reads were mapped to the human genome using Tophat (version 2.1.0) [48]. Reads aligned to the human genome were counted and quantified by FPKM using Cufflinks version 2.21 [49]. We used FPKM as the expression level of a protein. We used the number of tissues, where the FPKM was not 0, as the expression width of a protein. To analyse the difference of expression levels, the FPKMs of different tissues were normalized using Cuffnorm [49].

### Statistical analyses

Spearman’s rank test was applied to test the correlation of two datasets. Mann-Whitney U test was used to test the difference of dN values between two groups of proteins. Fisher’s exact test was implemented to test the difference of SNPs on domains. K-means was performed to cluster proteins based on expression levels. All statistical tests were performed using the R statistical package.

## Additional files

Additional file 1: Figure S1. Degree distribution of human integrated protein–protein interaction network. Degree (k), number of links connected to each protein; P(k), probability that a node has k links. (TIF 525 kb)
Additional file 2: Figure S2. Distribution of the variation rate of hub-non-bottlenecks and non-hub-bottlenecks using 60,706 humans dataset. (TIF 1206 kb)
Additional file 3: Figure S3. Distribution of the variation rate of hub-non-bottlenecks and non-hub-bottlenecks excluding difference of expression levels. (TIF 1138 kb)
Additional file 4: Figure S4. Distribution of the variation rate of hub-non-bottlenecks and non-hub-bottlenecks using HPRD interactions. (TIF 1311 kb)
Additional file 5: Figure S5. Distribution of the variation rate of hub-non-bottlenecks and non-hub-bottlenecks by using different cut-offs for hubs and bottlenecks. a) The cut-off is 1%. b) The cut-off is 5%. c) The cut-off is 10%. d) The cut-off is 15%. e) The cut-off is 30%. f) The cut-off is 40%. (TIF 2085 kb)

## Abbreviations

dN: Density of non-synonymous SNPs
PPI: Protein-protein interaction
SNPs: Single nucleotide polymorphisms
