# Supplementary figures and images for: Differential variation patterns between hubs and bottlenecks in human protein-protein interaction networks
Source: BMC Evol Biol. 2016 Dec 1;16:260. doi: 10.1186/s12862-016-0840-8 (PMC5131443; doi:10.1186/s12862-016-0840-8)

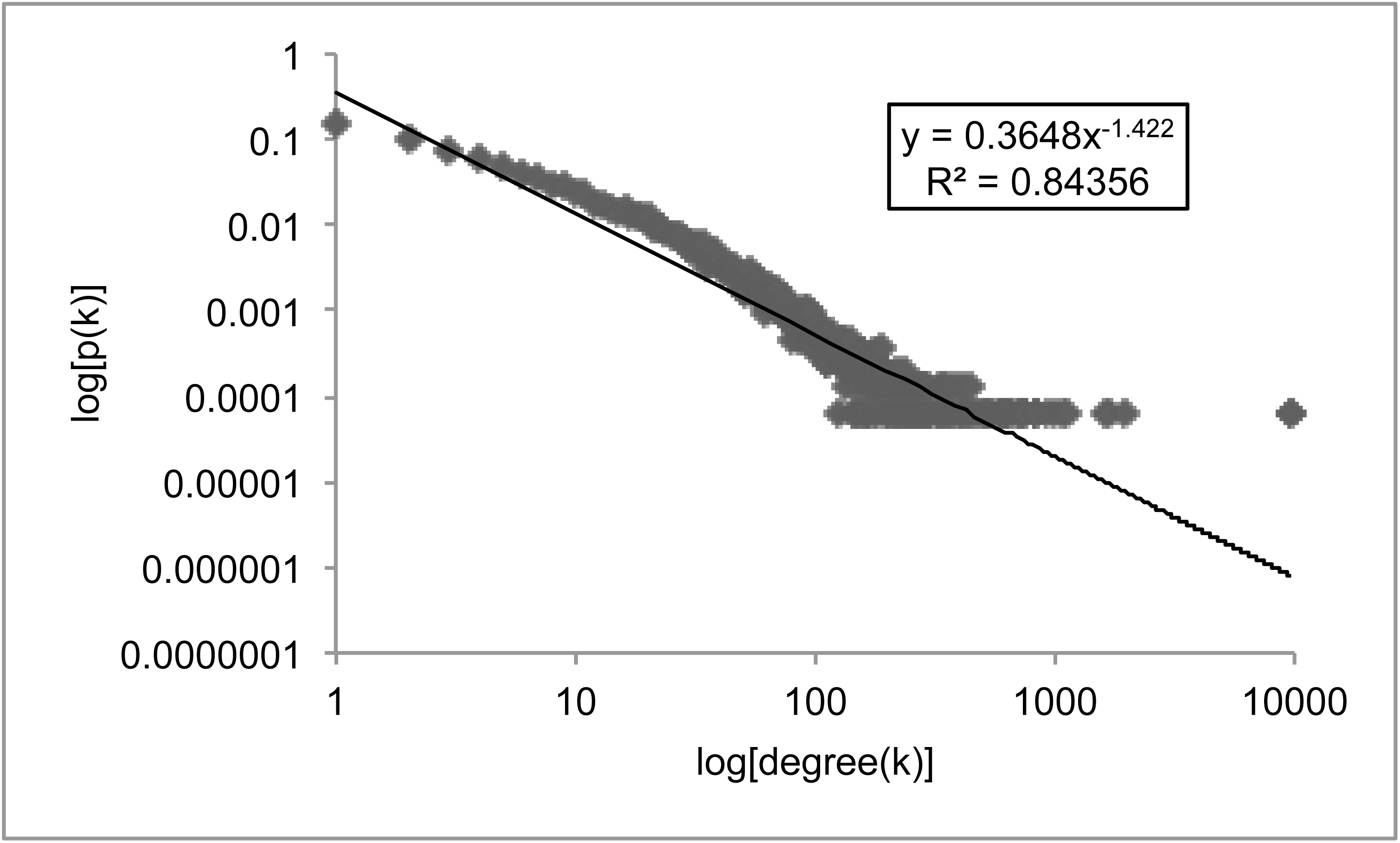

Supplement: Additional file 1: Figure S1. — Degree distribution of human integrated protein–protein interaction network. Degree (k), number of links connected to each protein; P(k), probability that a node has k links. (TIF 525 kb) [file 12862_2016_840_MOESM1_ESM.tif]

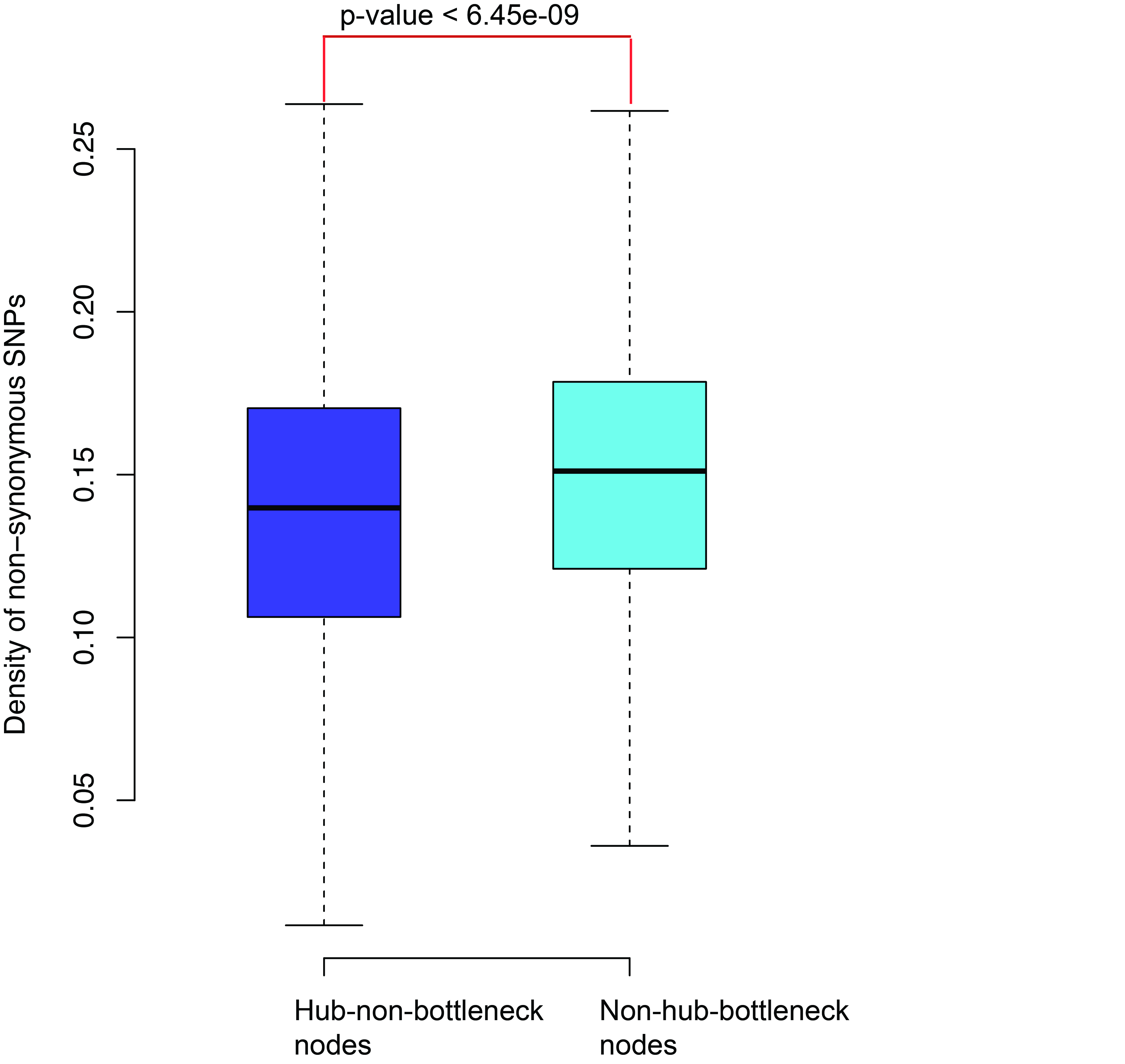

Supplement: Additional file 2: Figure S2. — Distribution of the variation rate of hub-non-bottlenecks and non-hub-bottlenecks using 60,706 humans dataset. (TIF 1206 kb) [file 12862_2016_840_MOESM2_ESM.tif]

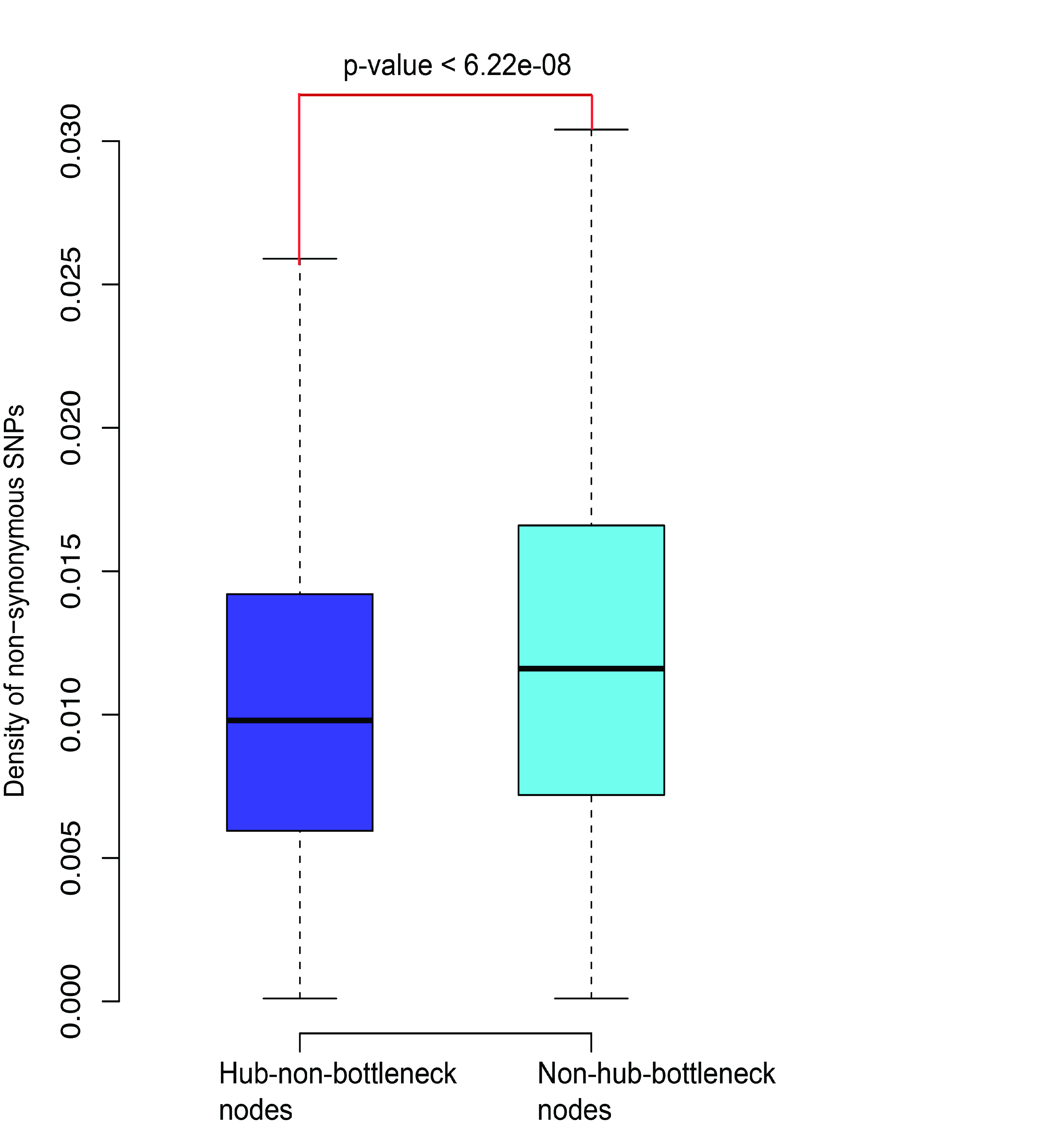

Supplement: Additional file 3: Figure S3. — Distribution of the variation rate of hub-non-bottlenecks and non-hub-bottlenecks excluding difference of expression levels. (TIF 1138 kb) [file 12862_2016_840_MOESM3_ESM.tif]

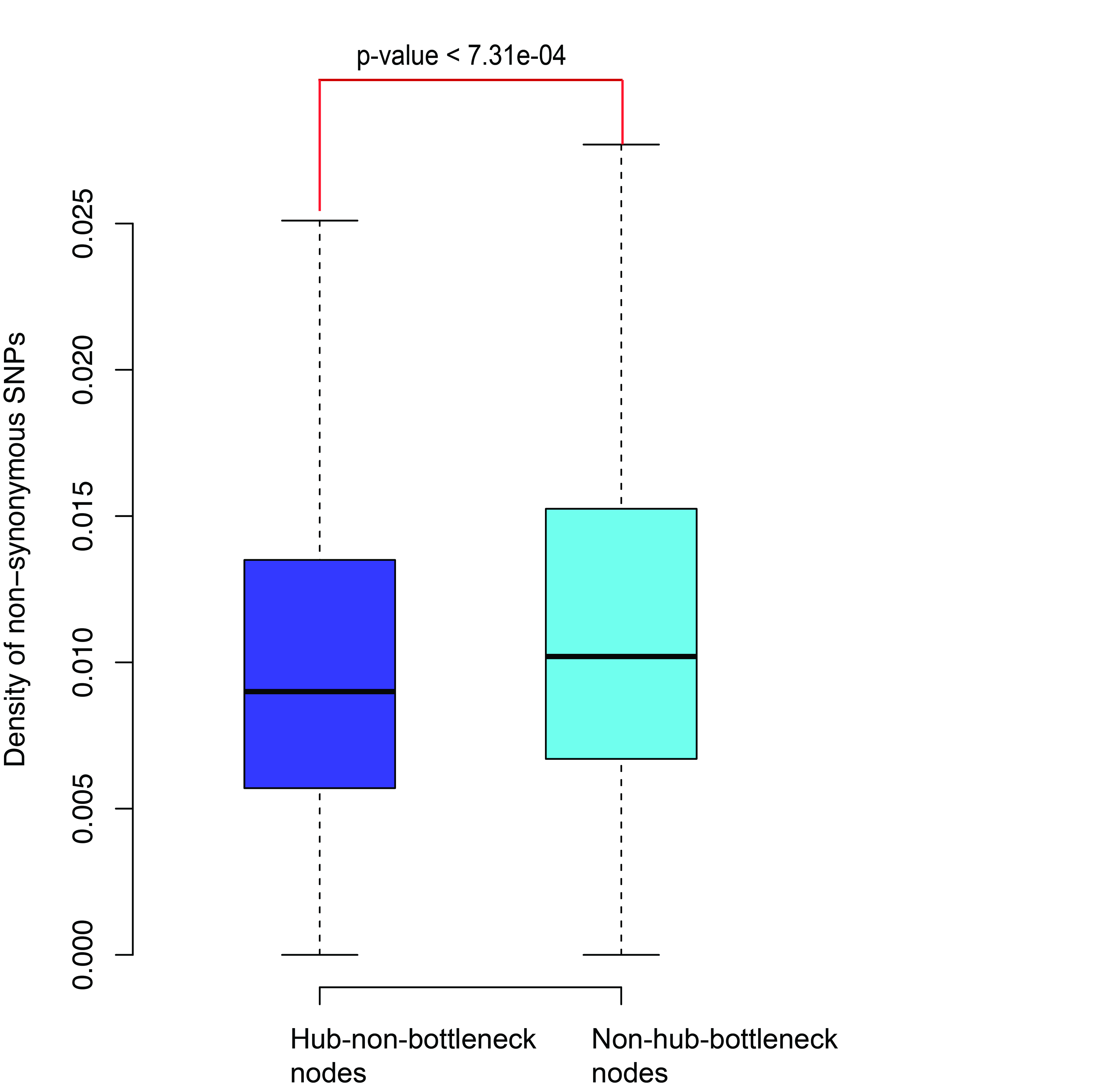

Supplement: Additional file 4: Figure S4. — Distribution of the variation rate of hub-non-bottlenecks and non-hub-bottlenecks using HPRD interactions. (TIF 1311 kb) [file 12862_2016_840_MOESM4_ESM.tif]

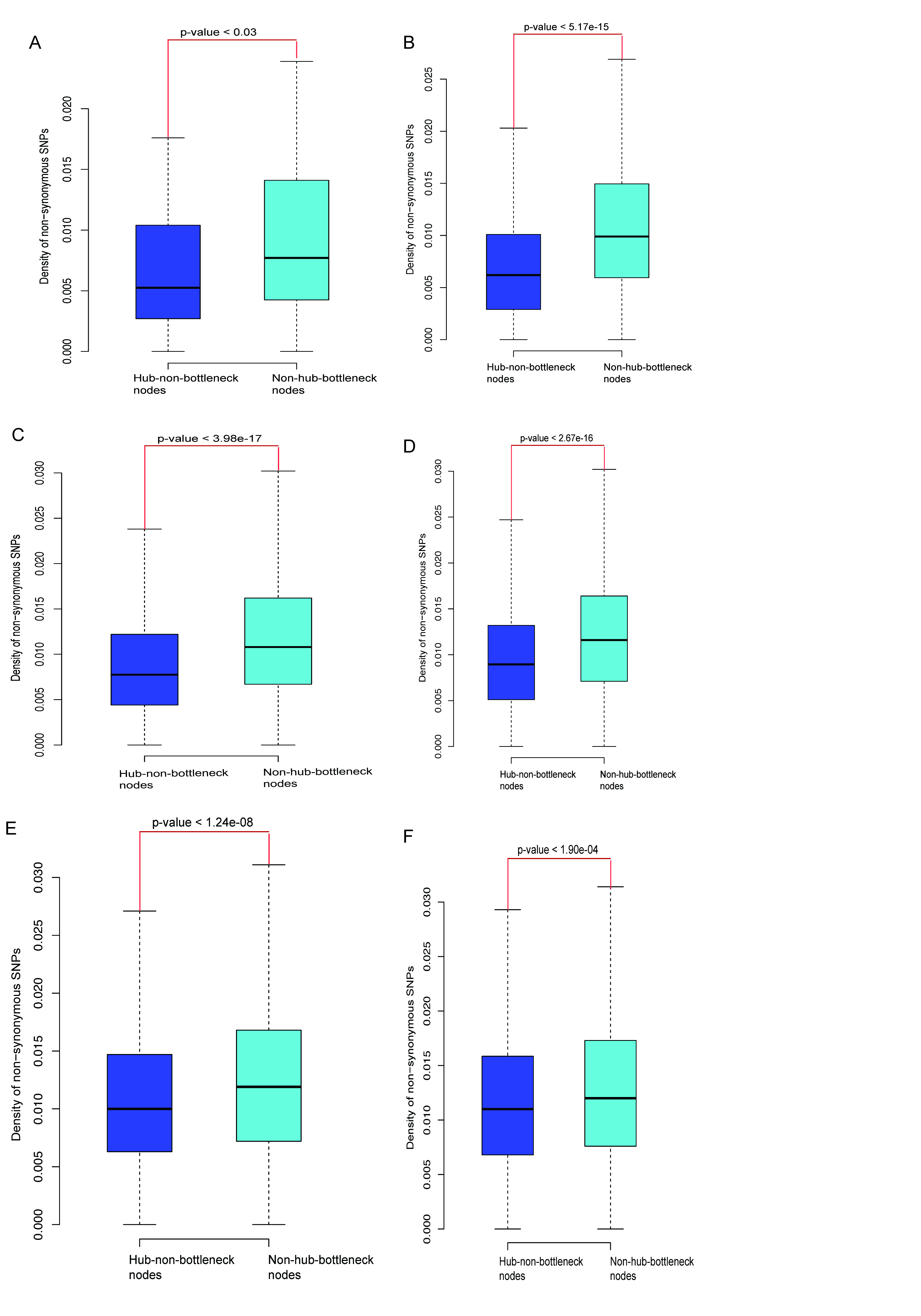

Supplement: Additional file 5: Figure S5. — Distribution of the variation rate of hub-non-bottlenecks and non-hub-bottlenecks by using different cut-offs for hubs and bottlenecks. a) The cut-off is 1%. b) The cut-off is 5%. c) The cut-off is 10%. d) The cut-off is 15%. e) The cut-off is 30%. f) The cut-off is 40%. (TIF 2085 kb) [file 12862_2016_840_MOESM5_ESM.tif]
